# Serum Levels of Mitochondrial and Microbial Metabolites Reflect Mitochondrial Dysfunction in Different Stages of Sepsis

**DOI:** 10.3390/metabo9100196

**Published:** 2019-09-20

**Authors:** Natalia Beloborodova, Alisa Pautova, Aleksandr Sergeev, Nadezhda Fedotcheva

**Affiliations:** 1 Federal Research and Clinical Center of Intensive Care Medicine and Rehabilitology, Petrovka str., 25-2, 107031 Moscow, Russia; alicepau@mail.ru (A.P.); sergeev_sklif@mail.ru (A.S.); 2 Institute of Theoretical and Experimental Biophysics, Russian Academy of Sciences, Institutskaya str., 3, Pushchino, 142290 Moscow Region, Russia; nfedotcheva@mail.ru

**Keywords:** sepsis, mitochondrial dysfunction, GC–MS, succinic acid, fumaric acid, itaconic acid, aromatic microbial metabolites, phenylcarboxylic acids, acidosis, succinate dehydrogenase

## Abstract

Mechanisms of mitochondrial dysfunction in sepsis are being extensively studied in recent years. During our study, concentrations of microbial phenolic acids and mitochondrial metabolites (succinic, α-ketoglutaric, fumaric, itaconic acids) as indicators of sepsis and mitochondrial dysfunction, respectively, are measured by gas chromatography–mass spectrometry (GC–MS) in the blood of critically ill patients at the early and late stages of documented sepsis. The increase in levels of some phenylcarboxylic (phenyllactic (PhLA), *p*-hydroxyphenylacetic (*p*-HPhAA), *p*-hydroxyphenyllactic (*p*-HPhAA)) acids (PhCAs), simultaneously with a rise in levels of mitochondrial dicarboxylic acids, are mainly detected during the late stage of sepsis, especially succinic acid (up to 100–1000 µM). Itaconic acid is found in low concentrations (0.5–2.3 µM) only at early-stage sepsis. PhCAs in vitro inhibits succinate dehydrogenase (SDH) in isolated mitochondria but, unlike itaconic acid which acts as a competitive inhibitor of SDH, microbial metabolites most likely act on the ubiquinone binding site of the respiratory chain. A close correlation of the level of succinic acid in serum and sepsis-induced organ dysfunction is revealed, moreover the most significant correlation is observed at high concentrations of phenolic microbial metabolites (PhCAs) in late-stage sepsis. These data indicate the promise of such an approach for early detection, monitoring the progression of organ dysfunction and predicting the risk of non-survival in sepsis.

## 1. Introduction

Mitochondrial dysfunction is a feature of many pathologies, including sepsis. Today, sepsis is defined as a life-threatening organ dysfunction caused by a dysregulated host response to infection. It is assumed that the mitochondrial dysfunction in sepsis is caused by the overproduction of cytokines, reactive oxygen species (ROS) and NO, which act directly or indirectly as inhibitors of some enzymes and complexes of the respiratory chain [1,2,3,4]. Among damaging factors, there are also local hypoxia caused by disturbances in the oxygen supply to organs, the exhaustion of the antioxidant protection system, and others. Moreover, there is evidence indicating that the multi-organ dysfunction syndrome accompanying sepsis is related to the mitochondrial dysfunction [1,3].

More specific mechanisms that cause dysfunction of the mitochondria at inflammation and sepsis have not been identified. The problem has become particularly relevant due to the discovery of a specific metabolite in mitochondria—itaconic acid, which is formed in the tricarboxylic acid cycle (TCA) in immune blood cells only under the action of bacterial lipopolysaccharides (LPS). Itaconic acid is synthesized by cis-aconitate decarboxylase, which is induced in response to inflammatory stimuli or infection [5,6,7,8,9]. Recently, a new pathway of succinic acid accumulation has been found. It also is induced by bacterial LPS and accompanied by a 30-fold increase of succinic acid concentration in macrophages [10,11,12]. Succinic acid is involved in the immune reactions by stimulating the selective production of pro-inflammatory cytokines, in particular, IL-1β, and activation of hypoxia-inducible factor HIF-1α [10,11]. The major effect of itaconic acid on cellular metabolism during macrophage activation is attributed to the inhibition of succinate dehydrogenase, although itaconic acid induces other effects, including anti-inflammatory and anti-bacterial ones.

Along with LPS, bacteria produce metabolites which can be accumulated in the blood at the acute stage of bacteremia and influence the tissues and organs at subsequent stages of the pathological process. Their role has been studied extensively only recently [13,14,15,16]. The data on the effects of microbial metabolites on mitochondrial functions relate mainly to their role in the development of renal and heart failure. Previously, microbial metabolites mainly were considered as uremic toxins. It was shown that indoleacetate, indoxyl sulfate, and phenylacetate inhibited SDH and the respiration of the kidney mitochondria by more than 20% at the concentration of these toxins in the range from 1 to 2 mM [17].

Currently, it is known that some microbial metabolites participate in the regulation of inflammatory responses and gene expression, affect membrane permeability and the blood–brain barrier [18,19,20]. Although microbial metabolites are necessary for normal metabolism and homeostasis of the body; at high concentrations they exhibit toxic effects [21,22]. This applies to all groups of microbial metabolites, including short chain fatty acids, methylamines, indolic and phenolic derivatives. The microbial metabolites, hyperproduction of which is observed in sepsis, are of the most interest.

Previously we have shown the most significant role of such microbial metabolites as PhCAs among many groups of studied low-molecular compounds in serum samples of septic patients. PhCAs are the products of degradation of phenylalanine, tyrosine and polyphenols by various bacteria from microbiota, including causative agents of sepsis. These compounds affect the mitochondrial functions and ROS production in liver mitochondria and neutrophils [13,14]. The effect of the PhCAs on mitochondria was enhanced by the acidosis, oxidative stress and substrates deficit, i.e., factors associated with sepsis [23].

Some PhCAs—PhLA, *p*-HPhAA, and *p*-HPhLA—have been proposed as new quantitative criteria for assessing bacterial load [24] as they are produced by *Staphylococcus aureus*, *Klebsiella pneumonia*, *Acinetobacter baumanii*, *Pseudomonas aeruginosa*, *Escherichia coli*, etc. [25,26]. The highest levels of these PhCAs are detected in the serum of patients with multiple organ dysfunction syndrome in sepsis [26,27,28]. These metabolites can come from both sources: the local focus of infection and intestinal bacteria [26,29,30]. Critical conditions are known to be accompanied by intestinal failure, which leads to a reduction in bacterial diversity and an accumulation of mostly gram-negative microorganisms, which are the main producers of the PhCAs [31]. The sum of three PhCAs reflects the disease severity as do the conventional scales Acute Physiology and Chronic Health Evaluation II (APACHE II) and Sequential (Sepsis-related) Organ Failure Assessment (SOFA) [27,28]. The PhCA concentrations are demonstrated to increase in dynamics in non-survived patients while they decrease gradually in critical surviving patients [32].

Since bacterial metabolites are linked to mitochondrial functions [16], in the present study we evaluate the concentrations of microbial PhCAs as indicators of sepsis simultaneously with mitochondrial metabolites as indicators of mitochondrial dysfunction in the blood serum of septic patients by GC–MS. Along with the conventional metabolites of the Krebs cycle, the possible presence of itaconic acid in the blood of patients also is evaluated. This metabolite was recently shown to inhibit SDH causing the succinic acid accumulation [8]. Although an attempt to test itaconic acid as a sepsis biomarker has already been made, it was not detected in the blood of patients with pneumonia-related sepsis [9]. During this study, we also try to detect itaconic acid in the blood of patients with abdominal sepsis. The influence of the PhCAs is compared with the effect of itaconic acid on the activity of SDH in vitro.

## 2. Results

### 2.1. Mitochondrial Metabolites in Blood Serum of Septic Patients

Succinic and fumaric acids were found in all 103 studied serum samples. Found in the control group, the levels of these two mitochondrial metabolites were relatively stable: succinic acid median (Me) was 22 (interquartile range (IQR) 15–26) µM, fumaric Me was 1.8 (1.3–2.4) µM (Table 1).

Discovered in serum samples taken from patients of Group I (early stage of sepsis), the levels of succinic acid varied widely (Table 1): from below 10 µM (in 36 samples, 75%) to medium 11–30 µM (in 10 samples, 21%) or to very high >100 µM in two cases (4%). The highest and the lowest values are not demonstrated in Table 1 as they are out of IQR (25–75%). The serum levels of fumaric acid at an early stage of sepsis were less than 10 µM and, in most cases (in 44 samples, 92%), did not exceed 4.4 µM. The correlation between the concentration of succinic acid and the SOFA score (*r* = 0.38) was revealed (Table 2).

Considering serum samples taken from patients of Group II (late stage of sepsis), the levels of succinic acid also varied widely (Table 1): from below 10 µM (in 14 samples, 40%), 11–30 µM (in 9 samples, 26%) to very high >100 µM (in 11 samples, 31%). The concentrations of fumaric acid were detected mostly at the levels below 10 µM (in 28 samples, 80%); in 7 samples, 20%, the levels of fumaric acids varied from 10 to 80 µM. The highest and the lowest values are not demonstrated in Table 1 as they are out of IQR (25–75%). There were close correlations of fumaric acid with succinic acid and the SOFA score in Group II: *r* = 0.74 and 0.55, respectively (Table 2).

Thus, succinic and fumaric acids were found in septic patients of Group II at significantly higher concentrations than in patients of Group I (*p < 0.01*) (Table 1). Fumaric acid closely correlated with succinic acid only in samples of late sepsis.

α-Ketoglutaric acid was found in healthy controls only in 1 of 20 cases (5%). Conversely in sepsis, α-ketoglutaric acid was revealed in more than half of patients: in 37 from 48 (77%) samples in early sepsis and in 16 from 35 (45%) samples in late sepsis.

Itaconic acid was not detected in healthy controls but was found in 5 samples of Group I. Serum concentrations of itaconic acid were within the range of 0.5–2.3 µM (Table 1).

### 2.2. Aromatic Microbial Metabolites in the Blood Serum of Septic Patients

Healthy controls (Group III) were characterized by low (less than 2 µM) levels of all PhCAs: benzoic acid (BA), phenylpropionic acid (PhPA), PhLA, *p*-HPhLA which were described in our previous paper [24].

Regarding serum samples from Group I, the following PhCAs were detected: BA, PhPA (traces), PhLA, *p*-HPhAA and *p*-HPhLA. The concentrations of three “sepsis-associated” PhCAs (PhLA, *p*-HPhAA and *p*-HPhLA) and their sum (Sum3) were, on average, 10 times greater than in the healthy control (Group III). The main contribution to the amount of Sum3 was made by *p*-HPhAA: Me 2.6 (1.0–8.6) µM. The concentrations of PhLA and *p*-HPhLA were: Me 1.1 (0.8–1.8) µM and 2.7 (1.3–6.0) µM, respectively.

Discussing serum samples taken from patients of Group II, the following PhCAs were detected: BA, PhLA, *p*-HPhAA and *p*-HPhLA. The concentrations of three “sepsis-associated” PhCAs and Sum3 were the highest among the all serum samples in our study and were, on average, 30 times greater than in the healthy control (Group III). The main contribution to the amount of Sum3 was made by *p*-HPhLA: Me 13.9 (1.1–28.9) µM. The concentrations of PhLA and *p*-HPhAA were Me 0.8 (0.4–2.2) µM and 1.1 (0.3–5.8) µM, respectively.

Close direct correlations of microbial and mitochondrial metabolites in serum samples of early and late sepsis were revealed (Table 2).

It is important to note that the results of the correlation analysis indicated the presence of a direct relationship between the severity of organ dysfunction (value of the SOFA score) and mitochondrial and microbial metabolites in blood. Regarding Group II, the highest correlation coefficient was observed between the serum levels of *p*-HPhLA and SOFA (*r* = 0.84) as well as between microbial and mitochondrial metabolites (*r* > 0.5). The late stage of sepsis will be examined more thoroughly later since, at this stage, the organ dysfunction often became irreversible and resulted in the death of patients.

### 2.3. Features of the Results in the Late Sepsis Group

#### 2.3.1. Microbial and Mitochondrial Metabolites in Groups II–III

**Figure 1:**
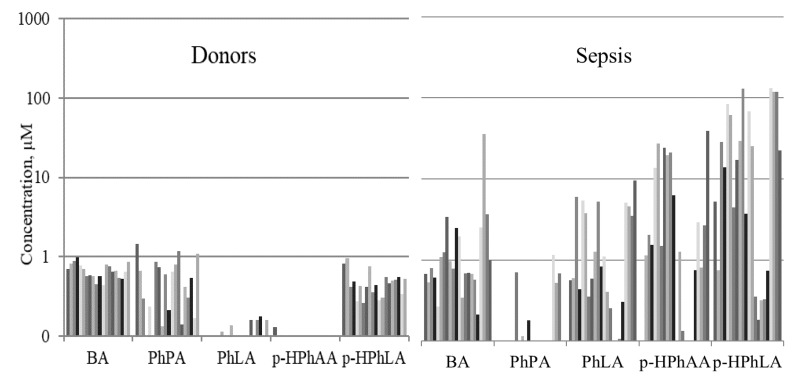
Concentrations of phenylcarboxylic acids (µM) in the serum samples of healthy controls (*n* = 20) and patients with late-stage sepsis (*n* = 22). Data is shown on a log scale with a base of 10.

We demonstrate the results of the simultaneous determination of the microbial and mitochondrial metabolites and lactate in the same serum samples of patients with late sepsis (*n* = 22) of Group II in comparison with healthy controls (Group III, *n* = 20). The concentrations of aromatic metabolites (BA, PhPA, PhLA, *p*-HPhAA and *p*-HPhLA) in the blood of healthy controls and septic patients are shown for each individual subject in Figure 1 (data is shown on a log scale with a base of 10). The concentrations of the PhCAs varied from less than 0.5 to 1.4 μM in the blood of healthy controls, while in septic patients the level of the PhCAs increased several (BA, PhLA) and even tens of times (*p*-HPhAA and *p*-HPhLA) (Figure 1).

**Figure 2:**
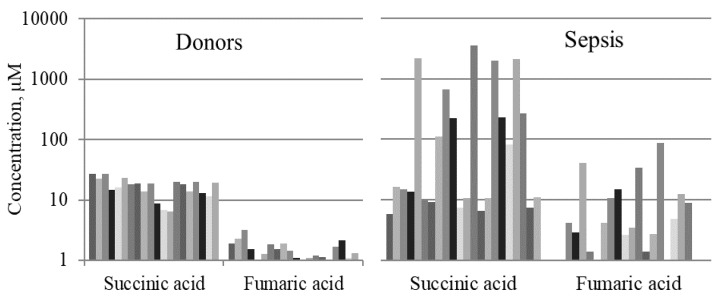
Concentrations of mitochondrial metabolites (µM) in the serum samples of healthy controls (*n* = 20) and patients with late sepsis (*n* = 22). Data is shown on a log scale with a base of 10.

The concentrations of succinic and fumaric acids in the blood of healthy controls and samples of septic patients are shown for each individual subject in Figure 2 (data is shown on a log scale with a base of 10).

It is noteworthy that in patients with late-stage sepsis (Group II) α-ketoglutaric acid, which is the “precursor” of succinic acid in the cycle of TCA, was often detected (in 41% cases), unlike the other Groups. This fact can be interpreted as inhibition of metabolic processes and deepening of mitochondrial dysfunction, but this situation requires further verification.

#### 2.3.2. Lactate, Mitochondrial and Microbial Metabolites

**Figure 3:**
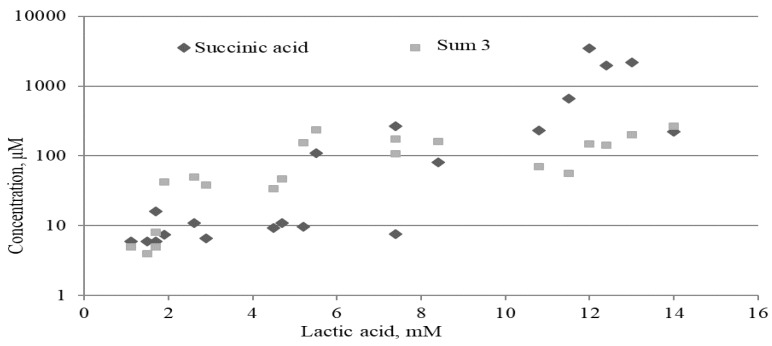
Comparison of concentrations of mitochondrial and microbial metabolites in relation to lactate levels obtained in the serum samples of the patients with late-stage sepsis in Group II (*n* = 22). The concentrations of three PhCAs and succinic acid are shown on a log scale with a base of 10.

Since the concentration of lactate in the blood remains one of the main indicators of sepsis, we compared simultaneously, in the same serum samples from septic patients, the concentration of lactic, succinic acids and Sum3. The lactate concentrations were 5.4 (2.4–11.0) mM. It turned out that higher concentrations of succinic acid and PhCAs corresponded to higher levels of lactic acid (Figure 3). High correlations were revealed between the levels of lactic and succinic acids (*r* = 0.86), lactic acid and Sum3 (*r* = 0.83), and succinic acid and Sum3 (*r* = 0.65). The significance of the revealed results in Group II indicates the importance of their verification on a larger group of patients.

### 2.4. Experimental Studies on Mitochondria: Effect of Phenylcarboxylic and Itaconic Acids on the Activity of Succinate Dehydrogenase

**Figure 4:**
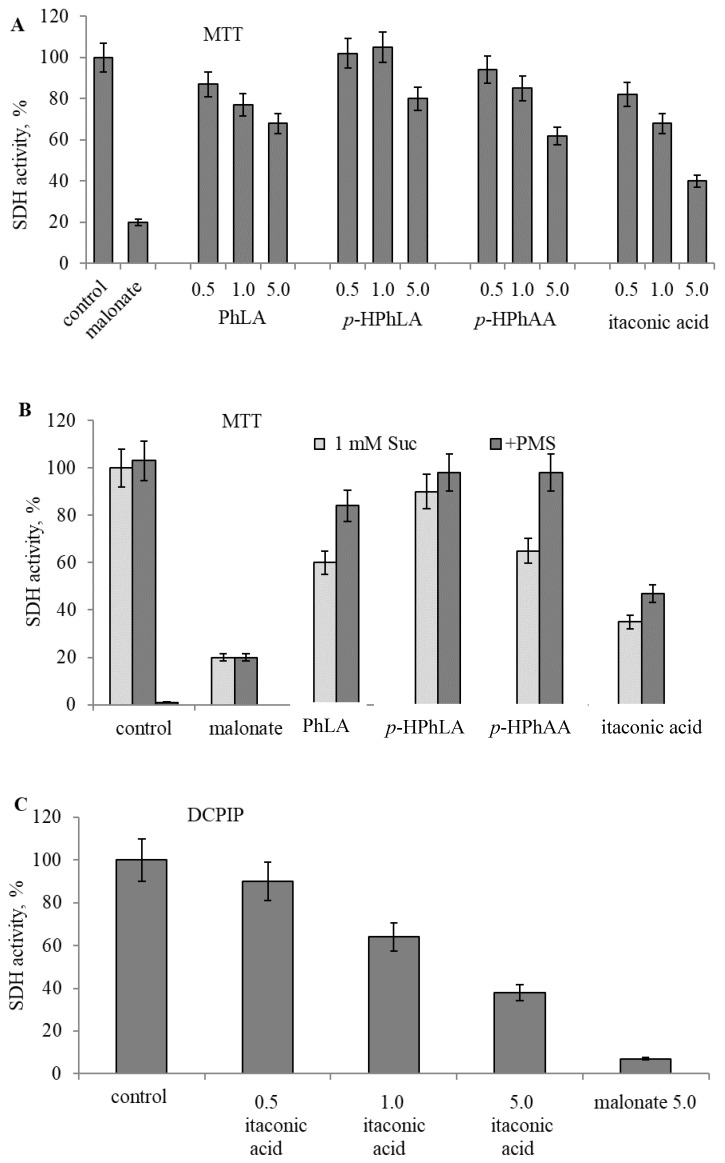

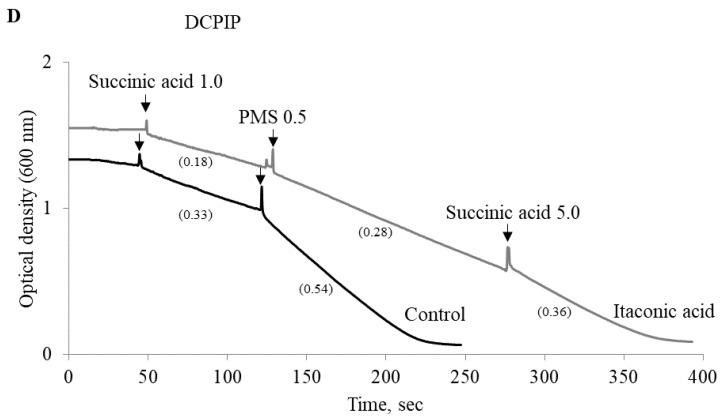
Influence of PhLA, *p*-HPhLA, *p*-HPhAA and itaconic acid at a 0.5–5.0 mM concentration range (indicated under the columns) on SDH activity after the 10 min. incubation with rat liver mitochondria measured by MTT reduction (**A**); SDH activation by phenazine methosulfate (0.5 mM PMS) after inhibition by PhLA, *p*-HPhLA, *p*-HPhAA and itaconic acid at a concentration for every compound of 5.0 mM (**B**); SDH inhibition by itaconic acid (0.5–5.0 mM) in comparison with malonate (5.0 mM) measured by DCPIP reduction (**C**) and influence of PMS and succinate excess (5.0 mM) on the DCPIP reduction rate during oxidation of succinate (1 mM) in the presence of 5 mM itaconic acid; the rate of DCPIP reduction, Δ/min, is indicated in parentheses (**D**) (*n = 5, P = 0.95*).

Previously, when investigating the role of PhCAs in the regulation of mitochondrial functions, we have shown that BA, cinnamic, phenylacetic (PhAA) and PhPA had a toxic effect in low concentrations (50–200 µM), while all other PhCAs acted only at concentrations of 1 mM and above [14]. Based on the assumption that they can be produced in high concentrations in the loci of lesions, we examined the effect of PhLA, *p*-HPhAA and *p*-HPhLA, whose concentrations rise in sepsis, on the activity of SDH in mitochondria. We compared their effects with the action of itaconic acid, which also inhibits SDH only at high concentrations (1–10 mM) [9]. The activity of SDH was estimated spectrophotometrically from the reduction of the electron acceptor methyl thiazolyl tetrazolium (MTT) or dichlorophenolindophenol (DCPIP). Since a substrate can protect an enzyme against inhibition, as it was shown for a number of dehydrogenases, the MTT reduction was assessed after the preliminary incubation of mitochondria with itaconic or PhCAs in the absence of an oxidation substrate. These conditions led to the inhibition of SDH by all compounds tested, which was not observed in the control when preincubation was carried out with succinic acid. The changes in SDH activity by the influence of the PhCAs and itaconic acid in the concentration range of 0.5–5.0 mM are shown in Figure 4A. Activity of SDH was measured by the reduction of MTT (Figure 4A,B) and by the rate of DCPIP reduction (Figure 4C,D).

At a concentration of 0.5–1.0 mM, they exhibited a weak inhibition, while at 5.0 mM, pronounced inhibition (from 20% to 50%) was observed for all compounds. The specificity of the reduction of MTT by SDH was confirmed by the complete inhibition of the acceptor reduction in the presence of malonate, a specific inhibitor of SDH.

While PhCAs and itaconic acid had an inhibitory effect on SDH, significant differences in the sites of their actions were found using the intermediate electron carrier PMS. It almost completely eliminated the inhibition induced by PhCAs and caused a slight activation in the case of itaconic acid (Figure 4B). PMS had no effect on the inhibition by malonate.

Similar changes also were observed with another specific electron acceptor, DCPIP. Itaconic acid, in this case, inhibited the rate of DCPIP reduction and this effect was abolished only slightly by PMS and more strongly by an excess of substrate (Figure 4C,D).

**Figure 5:**
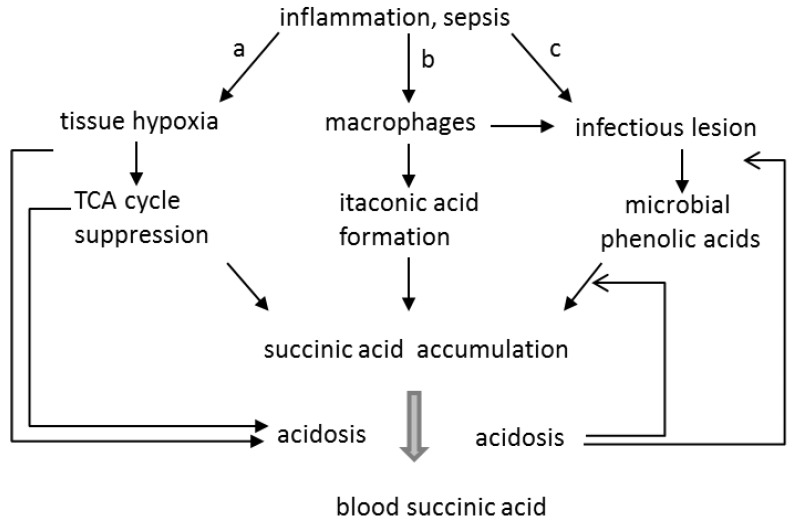
The combination of tissue and mitochondrial pathways contributing to an increase in the level of succinic acid in the blood of septic patients.

Summarizing the literature data and the results of our own studies, we can conclude that the increase in the level of succinate in the blood of septic patients could be the result of various mechanisms that are implemented in cells and mitochondria, which is schematically shown in Figure 5.

## 3. Discussion

A rise in the amount of microbial PhCAs and mitochondrial dicarboxylates, which depended on the severity of the disease and correlated with indicators of multi-organ dysfunction syndrome, was revealed. It can be assumed that an increase in the level of microbial metabolites specifically reflects microbial load, while the increase in the level of mitochondrial metabolites reflects mitochondrial dysfunction, which is one of the main signs of multi-organ dysfunction syndrome.

High levels of mitochondrial metabolites in the blood are attributed to a shift of the general metabolism, or to the inhibition of enzyme activity. An increase in the concentration of di- and tri-carboxylates in the blood is observed in pathologies associated with acidosis and hypoxia. Regarding patients with diabetic ketoacidosis and lactic acidosis, the concentration of succinic, α-ketoglutaric, and malic acids in the blood increased from 2 to 6 times [33]. Considering model experiments, extracellular succinic acid increased as a consequence of ischemia or hypoxia [33,34,35,36], reaching millimolar concentrations in the hypoxic area [37]. Normally, the succinic acid concentration in the blood varies from 15 to 26 μM [38,39], which corresponds to our data obtained from healthy controls (Me = 22 μM).

Inhibition of SDH by itaconic acid may be a sepsis-specific mode of succinate accumulation. Concerning our studies, itaconic acid was detected in the blood of septic patients only at an early stage of disease, in a small group of patients. It is possible that its appearance is one of the first manifestations of the onset of infection. Conversely, the excretion of itaconic acid happens at a low rate and, therefore, it cannot be detected in plasma [9]. It can be noted that macrophages are localized mainly in the loci of lesions, in this regard, where higher concentrations of itaconic acid can be expected. Moreover, at high concentrations, itaconic acid inhibits SDH not only in macrophages, but also in the mitochondria of non-immune cells [10] and, as shown in our work, it is an effective inhibitor of SDH in liver mitochondria.

The increase in the concentration of succinic, α-ketoglutaric, and fumaric acids in the blood suggests that the inhibition of dehydrogenases occurs in the part of the TCA cycle that involves the successive oxidation of α-ketoglutaric into succinic acid and then succinic into fumaric acid. It can be assumed that the accumulation of these metabolites is related to the inflow of α-ketoglutaric from glutamic and other amino acids. This assumption agrees with our data on the presence of α-ketoglutaric acid in 45–77% of cases of sepsis compared with its almost complete absence in healthy controls.

This pathway reflects an enhanced catabolism of proteins in acidosis, as it was previously proposed [40]. Moreover, the breakdown of total and myofibrillar proteins greatly increased during sepsis [41]. It is important that the enhancement of protein catabolism also can contribute to the production of PhCAs, derivatives of two aromatic amino acids—phenylalanine and tyrosine—by bacteria.

The total pool of PhCAs in norm is about 404 μM in the intestine and about 5.3 μM in the blood [14,29]. As it was shown in our data, the concentrations of individual PhCAs in the blood of septic patients increased to 50–150 μM and, according to the data of other authors, can be even higher. Thus, the level of PhAA in serum in chronic kidney disease reached 7 mM. At concentrations close to this value, PhAA inhibited the mitochondrial SDH activity by more than 20% in renal proximal cells [17]. Our results show that three “sepsis-associated” PhCAs at high concentrations inhibit SDH and can contribute to the accumulation of succinic acid. Unlike itaconic acid, which acts as an inhibitor of the catalytic subunit of SDH, PhCAs most likely act on the ubiquinone-binding site of the respiratory chain. However, they share a common mechanism of action associated with the interaction with thiol groups. The itaconic acid is a thiol-reactive metabolite which forms adducts with cysteine thiols [7]. PhCAs also act on thiol groups as their effect on mitochondria was removed by the thiol-reducing reagents [13,22]. The necessary factor facilitating the inhibition of SDH by these compounds is a deficit of oxidation substrate [23]. It can be assumed that an inhibition of SDH by PhCAs can occur in vivo, since these compounds, being monocarboxylates, easily penetrate into cells through the monocarboxylic acid transporter of an MCT-mediated transport system, which also transports pyruvate and amino acids [42,43]. Besides, as we found earlier, acidosis and deficiency of an oxidation substrate enhance the influence of microbial metabolites on mitochondrial functions [23]. The effective concentrations of PhCAs in such conditions are decreased by an order of magnitude. Therefore, it can be assumed that inhibition of SDH in late sepsis, accompanied by acidosis and debilitation, can occur at significantly lower concentrations of microbial metabolites.

The increase in the total pool of PhCAs in sepsis is made up of a significant increase in the concentrations of PhLA, *p*-HPhLA, and *p*-HPhAA. This result can be explained by interconversions between different PhCAs, which can occur in the course of redox-dependent reactions under both anaerobic and aerobic conditions. Under aerobic conditions, PhCAs can be transformed in the course of the sequential oxygenation to hydroxylated derivatives by facultative anaerobes, in particular, the sepsis-associated bacteria of different groups (gram-negative enterobacteria or gram-positive staphylococci). Besides, these and other PhCAs can accumulate in the tissue loci of lesions, as it occurs with mitochondrial metabolites that are concentrated mainly in the hypoxic area [29].

Thus, the observed variations in the concentrations of mitochondrial and microbial metabolites in blood may depend on the stage of sepsis and the extent of hypoxia in tissues, as well as disorders in different organs and tissues. Indeed, it was shown in model experiments on animals, these alterations do not occur simultaneously. Mitochondrial dysfunction in sepsis varies greatly, is specific to organs, and changes during sepsis as well as depends on the sepsis model used, time point of the examination, and tissue [44]. Following the induction of sepsis by the intraperitoneal injection of fecal slurry, the mitochondrial dysfunction occurred after 24 h in the liver and after 48 h in skeletal muscles [45], while after cecal ligation metabolic acidosis and ROS production were observed even in the first 3 h [46]. Such changes are probably summarized at the late stage of sepsis and manifest themselves in increased levels of certain metabolites observed in our studies. Regarding all cases of late sepsis, the increase in the level of mitochondrial metabolites correlated with an increase in the total pool of microbial PhCAs and coincided with the rise of lactate level in the blood. Although lactic acidosis remains one of the main indicators of sepsis [47,48], it is characteristic of many other pathologies. Blood succinic acid is more specific to the inflammatory syndrome, and the simultaneous presence of microbial metabolites in the blood can serve as a distinctive feature of sepsis.

Similar data were obtained for patients with hemorrhagic shock, in which a high concentration of succinic acid (up to 200–600 µM) in plasma correlated with the severity of damage and the risk of death. This increase in the concentration of succinic acid coincided with the rise of the lactate concentration (up to 15 mM) in the blood [49], as in our present study, which may indicate similar mechanisms of thanatogenesis in hemorrhagic and septic shock.

The main above-mentioned ways leading to an increase of succinic acid and microbial PhCA levels in the blood in sepsis are presented in Figure 5. Path “a” shows the role of ROS inducing the damage to the endothelium and hypoxia in tissues followed by the accumulation of succinic acid in mitochondria. Path “b” indicates the role of LPS stimulating the production of itaconic acid, an inhibitor of SDH, in macrophages. Path “c” demonstrates the area of infection with an increased production of microbial PhCAs, which act as SDH inhibitors. Figure 5 also shows the key role of acidosis in these processes. Acidosis enhances the production of PhCAs and their influence on mitochondrial functions [23], as well as facilitates the release of succinic acid into the blood from ischemic tissues [50].

The review of Stanzani, G. et al. [3] analyzes a number of studies confirming the important role of circulating factors in the pathogenesis of sepsis-induced organ dysfunction and emphasizes the need to search novel and sensitive biomarkers of mitochondrial damage and function. This study for the first time, to the best of our knowledge, revealed a close correlation of succinic acid and sepsis-induced organ dysfunction. This relationship was the most significant in the late stage of sepsis at a high concentration of phenolic microbial metabolites. More extensive studies are needed in future to confirm the feasibility of testing succinic acid in the blood as a target for assessing the degree of mitochondrial dysfunction in sepsis, while monitoring sepsis-associated microbial metabolites to control targeted therapy.

Taking place in the early stages of sepsis, the heterogeneity of serum succinic acid levels most likely is associated with the involvement of non-mitochondrial mechanisms of organ dysfunction. During septic shock, for example, a violation of the hormonal regulation of vascular tone by catecholamines also may be associated with a high level of phenolic metabolites, predominantly *p*-HPhAA [27]. Low levels of mitochondrial metabolites in the blood (lower than in healthy controls) may reflect another variant of the anomaly in the mitochondrial paths leading to reduced cellular metabolism and energy production. It is assumed that sepsis is one of the diseases in which mechanisms like hibernation play a role [3,51,52]. Such metabolic sleep is proposed to be interpreted as an adaptation mechanism—temporary dysfunction of cells for their survival in a critical situation. The study of hibernation as a natural phenomenon in some animals showed significant differences in the metabolism of mitochondria. The levels of endogenous metabolites of the TCA cycle in the liver, brown adipose tissue and brain of the arctic ground squirrels were estimated as possible indicators of mitochondrial processes. Metabolic depression manifested itself as inhibition at the initial stages of the TCA cycle. Succinic acid was not detected in any of tissues and serum lactate decreased five-fold in torpor in a temperature-dependent manner with a long period of persistence of stable concentration [53]. Thus, in sepsis, hibernation-type mechanisms may take place, but rather a transition from adaptive to maladaptive organ dysfunction occurs.

## 4. Materials and Methods

### 4.1. Reagents and Chemicals

2,3,4,5,6-D_5_-benzoic acid (internal standard, ≥99 atom % D, ≥99%), benzoic acid (BA, ≥99.5%), phenylpropionic acid (PhPA, ≥99%), phenyllactic acid (PhLA, ≥98%), 4-hydroxyphenylacetic acid (*p*-HPhAA, ≥98%), 4-hydroxyphenyllactic acid (*p*-HPhLA, ≥97%), 3,4-dihydroxybenzoic acid (internal standard, ≥98%), succinic acid (≥99%), α-ketoglutaric acid (≥98%), fumaric acid (≥99%), itaconic acid (≥99%), *N,O*-bis(trimethylsilyl)trifluoroacetamide (contains 1% trimethylchlorosilane, 99% *N,O*-bis(trimethylsilyl)trifluoroacetamide), hexane (≥97.0%) were obtained from Merck (Darmstadt, Germany); sulfuric acid, acetone, diethyl ether, sodium chloride were Laboratory Reagent grade and obtained from Khimreactiv (Staryy Oskol, Russia). All other reagents were from the Sigma–Aldrich Corporation (St. Louis, MO, USA).

### 4.2. Patients and Samples

Blood serum samples and clinical data according to the protocol were taken from seriously ill patients who were treated in ICUs in Moscow clinical hospitals. The approval of the local ethics committee was previously obtained (N 26/2019). During routine blood sampling in hospitalized patients for biochemical monitoring the residual serum was taken, frozen, and kept at −30 °C for the analysis of microbial and mitochondrial metabolites by GC–MS. The severity of organ dysfunctions in sepsis was confirmed by biochemical parameters, elevated levels of procalcitonin (2–140 ng/mL) and assessment of the SOFA score. The stages of sepsis—early or late—were allocated conditionally, depending on the timing of diagnosis. Three groups of serum samples were examined: early-stage sepsis; late-stage sepsis; healthy control.

Samples of Group I (the early stage of sepsis) were taken from patients (age 55 (14–65) years, 62.5% male) on the first day of diagnosis of sepsis and repeatedly during the process of intensive treatment. All cases of sepsis were documented in accordance with the criteria of the Third International Consensus on the Definition of Sepsis and Septic Shock (SEPSIS - 3) [54]. There were 8 patients with sepsis: severe injury (*n* = 3), pneumonia (*n* = 4) or abdominal infection (*n* = 1). The median values of the SOFA score on the days of blood sampling for the study of metabolites were 9 (7–12). The lactate level was >2 mM in all patients, Me = 4.2 mM. Seven of eight patients survived by the 28th day in the ICU; the mortality in this group was 12.5%. All patients were in a severe period of septic shock and were treated with extracorporeal detoxification methods and were examined in dynamics. Serum samples were collected repeatedly before and after the hemodiafiltration or hemosorption within a few days until the decrease in the symptoms of the life-threatening stage or in death. Thus, 48 serum samples from 8 patients were studied in this group.

Samples of Group II (the late stage of sepsis) were taken from extremely severe, almost terminal, mostly elderly patients (age 62 (48–68) years, 70% male) with sepsis. These septic patients were admitted to the ICU after 2–4 weeks due to surgical complications, with clinical signs of progressive organ dysfunction and having an unfavorable prognosis. Most patients (18/22, 82%) in this group had abdominal sepsis. Pathological processes in the abdomen (cancer, mesenteric thrombosis, gastric ulcer, calculous cholecystitis, enterocolitis, liver injury, pancreatic necrosis) had a complicated course and all signs of sepsis due to the development of peritonitis (6), intestinal obstruction (2); against the background of the underlying disease, severe pneumonia (6), gastrointestinal bleeding (3) and other complications developed. Some patients (4/22, 18%) had other reasons for admission to the ICU: pulmonary embolism (2), acute cerebrovascular accident (1), acute myocardial infarction (1), but then they developed severe pneumonia with multiple organ failure, impaired hemodynamics, which met the criteria for sepsis. Nine patients had surgery, 4 of them repeatedly. Despite comprehensive treatment in the ICU, including detoxification, antibiotic therapy, mechanical ventilation, and vasopressors, 17 of 22 (77%) of patients in this group died in the next few days. The median values of the SOFA score on the days of blood sampling for the study of metabolites were 11 (9–14). Serum samples were taken in dynamics every 2–3 days, or until death. Thus, 35 serum samples from 22 patients were studied in this group.

Samples of Group III (healthy control) were taken from adult healthy donors (*n* = 20) in Dmitry Rogachev National Research Center of Pediatric Hematology, Oncology and Immunology (Moscow, Russia). Healthy controls did not have chronic liver and kidney diseases or clinical signs of acute inflammation; they all had a normal body temperature and normal levels of leukocyte, platelet count, hemoglobin, bilirubin, urea and creatinine.

### 4.3. Serum Sample Preparation and GC–MS Analysis

All told, 103 blood serum samples were investigated using the GC–MS method. Samples were defrosted at room temperature prior to use. All GC–MS analyses were performed on a Trace GC 1310 gas chromatograph equipped with an ISQ LT mass spectrometer using the capillary column TR-5ms (95% poly(dimethylsiloxane) + 5% phenyl polysilphenylene-siloxane phase, 30 m × 0.25 mm, df = 0.25 µm) obtained from Thermo Scientific (Thermo Electron Corporation, USA). The column flow was constant at 1.5 mL min^−1^ with helium as the carrier gas, split 1:20. The GC analysis was performed in 25 min with a starting oven temperature of 80 °C (hold time 4 min) and a single ramp of 10 °C min^−1^ to 250 °C (hold time 4 min). The injector temperature was 200 °C and the injection volume was 1 µL. Full-scan mass spectra were recorded with an *m/z* range of 50–480 in the electron-impact mode at 70 eV, using Xcalibur 2.2 software. The MS source was 200 °C and the GC–MS interface was kept at 250 °C. Scan rate was 3 scans/s; cathode delay time 4 min; and cathode turn-off time 20 min.

The conditions of liquid–liquid extraction of the PhCAs were previously described [32]. Briefly, an aliquot (200 µL) of serum and aliquots (100 µL) of aqueous solution of internal standards (2,3,4,5,6-D_5_-benzoic and 3,4-dihydroxybenzoic acids with a concentration of 4 ng/µL) were diluted with 600 µL of distilled water. Solid sodium chloride (0.3–0.5 g) and concentrated sulfuric acid (15 µL) were added. An extraction with diethyl ether was carried out (2 × 1 mL). The ether extract was evaporated at 40 °C and derivatized with *N,O*-bis(trimethylsilyl)trifluoroacetamide (20 µL, 80 °C, 15 min). The solution with trimethylsilyl derivatives was cooled at 5 °C for 30 min, diluted with 100 µL of *n*-hexane, and 1 µL of the final solution was injected into the GC–MS system.

Trimethylsilyl derivatives of the PhCAs were identified using retention times and characteristic *m/z* values which were previously described [32]. Quantitative analyses were carried out using internal standards: 2,3,4,5,6-D_5_-benzoic acid for BA and PhPA; 3,4-dihydroxybenzoic acid for PhLA, *p*-HPhAA, and *p*-HPhLA. The concentrations of the PhCAs were calculated using the equations of linear functions.

Trimethylsilyl derivatives of succinic, fumaric, α-ketoglutaric and itaconic acids were identified using retention times and characteristic *m/z* values, described in Table 3. Quantitative analyses of succinic, fumaric and itaconic acids were carried out using 2,3,4,5,6-D_5_-benzoic acid as an internal standard according to the equations of linear functions.

### 4.4. Preparation of Rat Liver Mitochondria

Mitochondria were isolated from adult *Wistar* male rats. The study was conducted in accordance with the ethical principles formulated in the Helsinki Declaration on the care and use of laboratory animals. Manipulations were carried out by the certified staff of the Animal Department of the Institute of Theoretical and Experimental Biophysics (Russian Academy of Science) and approved by the Commission on Biomedical Ethics of ITEB RAS (N H 01/18). During the study, the animals were kept in wire-mesh cages at room temperature (22 °C) with a light/dark cycle of 12 h. Mitochondria from the liver of anesthetized animals were isolated using the standard method [14]. The liver was rapidly removed and homogenized in an ice-cold isolation buffer containing 300 mM sucrose, 1 mM EGTA, and 10 mM HEPES–Tris (pH 7.4). The homogenate was centrifuged at 600 *g* for 7 min at 4 °C and the supernatant fraction was then centrifuged at 9000 *g* for 10 min to obtain mitochondria. Mitochondria were washed twice in the above medium without EGTA and BSA. The final mitochondrial pellet was suspended in the washing medium to yield 60 mg protein/mL and kept on ice for analysis.

### 4.5. Determination of Succinate Dehydrogenase Activity from Reduction of Methyl Thiazolyl Tetrazolium and Dichlorophenolindophenol

An incubation medium (2 mL) containing 125 mM KCl, 20 mM HEPES, pH 7.4, 150 μM MTT, and the oxidation substrate were mixed with mitochondria (0.5 mg protein per mL) and incubated for 5 min as described earlier [55]. The examined samples of PhCAs were placed simultaneously in a series of spectrophotometric cuvettes. The reaction of acceptor reduction was initiated by addition of mitochondria. Pre-incubation with PhCAs in the absence of an oxidation substrate was carried out for 5 min, then the substrate and MTT were added and the incubation continued for the next 5 min. Subsequent to the incubation, mitochondria were lysed by Triton X-100 (20 μL of 10% solution) and optical density was recorded at 580 nm with an USB4000 spectrophotometer (Ocean Optic, USA).

The activity of SDH also was determined by the reduction of the electron acceptor DCPIP [56]. Mitochondria (0.5 mg protein/mL) were incubated in 2 mL of medium containing 125 mM KCl, 15 mM HEPES, pH 7.4 in the presence of 1 mM cyanide, 20 μL of 10% Triton X-100, 100 μM DCPIP. The DCPIP reduction reaction was induced by the addition of 5 mM succinic acid, further activated with 250 μM PMS, and the acceptor reduction rate was measured at a wavelength of 600 nm using an Ocean Optics USB4000 spectrophotometer.

### 4.6. Statistical Analysis

The clinical data are presented as median (Me) and interquartile range (IQR, 25–75%). Correlation analysis was performed using Spearman’s correlation coefficient, *p* < 0.05 was used as the level of significance. To assess the differences between small independent groups, we used the non-parametric Mann–Whitney U test. All data were analyzed using Microsoft Excel 2016.

The experimental data on mitochondria shown represent the means ± standard error of means or are the typical traces of five identical experiments with the use of different mitochondrial preparations.

## 5. Conclusions

Metabolic changes and mitochondrial dysfunction that occur in the body during sepsis usually lead to changes in the concentrations of mitochondrial metabolites not only in tissues, as it seemed before, but also in blood serum. To contrast to healthy controls, where the levels of succinic and fumaric acids in the blood are relatively stable, we observed changes in their concentrations from very low to extremely high in patients with sepsis, exceeding the norm 10–100 times. The highest levels of mitochondrial metabolites were more typical for patients in the late stage of sepsis with death. Increase in the concentrations of succinic acid simultaneously with PhCAs in the blood during sepsis is associated with lactic acidosis. Unlike other pathologies associated with hypoxia and acidosis, aromatic microbial metabolites (PhCAs) always are accumulated in the blood of patients with sepsis. A close direct correlation of serum concentrations of the PhCAs with mitochondrial succinic, fumaric acids and with a SOFA score of multi-organ dysfunction syndrome confirms the participation of these microbial metabolites in the development of mitochondrial dysfunction in sepsis.

## Figures and Tables

**Table 1:** Concentrations of aromatic microbial metabolites and mitochondrial metabolites in the serum of three groups of samples, Me (IQR 25–75%), µM.

Metabolite	Group I (Early Stage of Sepsis)	Group II (Late Stage of Sepsis)	Group III (Healthy Control)	*p _I, II_*	*p _II, III_*	*p _I, III_*
Aromatic Microbial Metabolites						
PhLA	1.1 (0.8–1.8)	0.8 (0.4–2.2)	<LOD *	*ns*	*<0.0001*	*<0.0001*
*p*-HPhAA	2.6 (1.0–8.6)	1.1 (0.3–5.8)	<LOD *	*ns*	*<0.0001*	*<0.0001*
*p*-HPhLA	2.7 (1.3–6.0)	13.9 (1.1–28.9)	0.7 (0.6–1.0)	*<0.05*	*<0.0001*	*<0.0001*
Sum3	7.4 (3.2–22.8)	15.9 (3.4–59.4)	0.8 (0.6–1.1)	*ns*	*<0.0001*	*<0.0001*
Mitochondrial Metabolites						
Succinic Acid	4.9 (4.0–13.7)	11.0 (7.4–169.8)	22.0 (15.5–26.2)	*<0.0001*	*ns*	*<0.0001*
Fumaric Acid	1.0 (0.9–2.7)	2.7 (0.9–6.3)	1.8 (1.3–2.4)	*0.002*	*ns*	*0.007*
Itaconic Acid	1.2 (0.5–2.3)	<LOD *	<LOD *	-	-	-
α-Ketoglutaric Acid	77% **	45% **	5% **	-	-	-

* the concentration is below the limit of detection (LOD); ** as α-ketoglutaric acid was determined qualitatively, data is demonstrated as the percentage of presence in each group.

**Table 2:** Correlations (*r*) of the SOFA score with serum levels of mitochondrial metabolites and phenylcarboxylic acids in septic patients (*p < 0.05*).

Group I (Early Stage of Sepsis)				Group II (Late Stage of Sepsis)			
Parameter	SOFA	Succinic Acid	Fumaric Acid	Parameter	SOFA	Succinic Acid	Fumaric Acid
SOFA	-	0.38	ns	SOFA	-	0.61	0.55
Succinic Acid	0.38	-	ns	Succinic acid	0.61	-	0.74
Fumaric Acid	ns	ns	-	Fumaric acid	0.55	0.74	-
PhLA	0.45	0.39	−0.29	PhLA	0.69	0.45	0.62
*p*-HPhAA	0.59	0.40	ns	*p*-HPhAA	0.57	ns	0.39
*p*-HPhLA	0.39	0.39	ns	*p*-HPhLA	0.84	0.54	0.68
Sum3	0.52	0.45	ns	Sum3	0.83	0.54	0.68

ns - not significant.

**Table 3:** Retention times, characteristic *m/z* values and equations of linear functions of trimethylsilyl derivatives of succinic, fumaric, α-ketoglutaric and itaconic acids.

Acid Derivatives	Retention Time, min	*m/z* Value	Range of Concentrations, µM	Linear Equation	*R^2^*
Succinic	10.67	247 and 172	0.5–70	y = 435 × x	0.9920
Fumaric	11.22	245 and 217	0.5–70	y = 31 × x	0.9988
α-Ketoglutaric	13.15	157 and 173	*	*	*
Itaconic	11.10	215 and 259	0.5–15	y = 481 × x	0.9886

* α-Ketoglutaric acid was determined qualitatively and its quantitative analysis is presumably limited to the conditions of sample preparation.

## Abbreviations

BA	benzoic acid
DCPIP	dichlorophenolindophenol
ICU	intensive care unit
IQR	interquartile range
GC-MS	gas chromatography-mass spectrometry
LPS	lipopolysaccharides
MTT	methyl thiazolyl tetrazolium
PhAA	phenylacetic acid
PhCA	phenylcarboxylic acid
PhPA	phenylpropionic acid
PhLA	phenyllactic acid
*p*-HPhAA	4-hydroxyphenylacetic acid
*p*-HPhLA	4-hydroxyphenyllactic acid
PMS	phenazine methosulfate
ROS	reactive oxygen species
SDH	succinate dehydrogenase
SOFA score	sequential organ failure assessment score
Sum3	sum of PhLA, *p*-HPhAA and *p*-HPhLA
TCA	tricarboxylic acid cycle
